# Providing insight in resilience and recovery by using fatigability and its development over time in older adults after hip fracture surgery

**DOI:** 10.1007/s41999-026-01514-x

**Published:** 2026-06-04

**Authors:** Jolijn Dijksterhuis, Marian Z. M. Hurmuz, Dieuwke van Dartel, Marloes Vermeer, René Melis, Johannes H. Hegeman

**Affiliations:** 1 Department of Trauma Surgery, Ziekenhuisgroep Twente, 7609 PP Almelo, The Netherlands; 2 Department of Biomedical Signals and Systems, University of Twente, 7500 AE Enschede, The Netherlands; 3 ZGT Academy, Ziekenhuisgroep Twente, 7609 PP Almelo, The Netherlands; 4 Reggeborgh Research Fellow, ZGT Academy, Ziekenhuisgroep Twente, 7609 PP Almelo, the Netherlands; 5 Department of Geriatrics, Radboud University Medical Center, PO Box 9101, NL-6500 HB Nijmegen, The Netherlands

**Keywords:** Hip fracture, Surgery, Older adults, Grip work, Recovery, Fatigue

## Abstract

**Purpose:**

Older hip fracture patients are often frail, complicating their recovery trajectories. Insight into patients’ resilience may help in getting more understanding of their predicted recovery and adjusting their treatment plan accordingly. This study aims to assess if fatigability, calculated with the Capacity to Perceived Vitality ratio (CPV), at baseline and its development over time, can provide insight into recovery and resilience at hospital discharge.

**Methods:**

In this prospective cohort study, patients were included from March 2022 to February 2024. Growth modelling was used to test whether there was a change in CPV over time during hospitalization and which covariables were associated with CPV. Subsequently, two logistic regression analyses were conducted to assess the role of CPV as a predictor of mobility at discharge (FAC score) and hospital complications (yes/no).

**Results:**

CPV did not significantly change over time (estimate = 0.99; 95%CI 0.90–1.10). At baseline, men had a factor 1.6 (*p* = 0.01) higher CPV than women and patients with a pre-fracture KATZ-ADL 6 score of 0 had a factor 2.1 (*p* = 0.003) higher CPV than patients with a pre-fracture KATZ-ADL 6 score of 2 or more. The logistic regression models showed that when the intercept of CPV increased, the odds of a dependent FAC score at discharge (*p* = 0.002) and of developing a complication (*p* = 0.037) decreased.

**Conclusion:**

CPV at baseline had predictive value for mobility at discharge and complications during hospitalization. Furthermore, there was no significant change in CPV over time, and the covariables gender and pre-fracture KATZ-ADL 6 mainly influenced the CPV at baseline.

## Key summary points

**Aim:**

This study aimed to assess whether combined grip work (GW) and self-perceived fatigue (SPF) (fatigability, calculated with the Capacity to Perceived Vitality ratio (CPV)) and its development over time, can provide insight into recovery and resilience in older adults after hip fracture surgery.

**Findings:**

The CPV at baseline seems to have a predictive value for mobility at discharge and complications during hospitalization, both of which are part of recovery. Furthermore, there was no significant change in CPV over time, and the covariables gender and pre-fracture KATZ-ADL 6 mainly influenced CPV at baseline.

**Message:**

CPV at baseline provided an important contribution to insight into resilience through predicting short-term hospital discharge outcomes such as mobility and complications.

## Introduction

A hip fracture is a common injury among older adults, including approximately 18,000 older hip fracture patients each year in the Netherlands [1]. The incidence of hip fractures is expected to increase in the future due to the aging population and longer life expectancy [2]. Older adults with a hip fracture are often frail due to their age and the presence of comorbidities, which makes their recovery complex. Additionally, the recovery after a hip fracture is unpredictable, as older adults often show different recovery trajectories [3]. This difference in recovery can depend on a variety of factors including age, gender, cognitive status, comorbidities, and pre-fracture functional status, all of which also play a significant role in influencing a person’s resilience [3, 4]. Resilience is defined as the ability to resist functional decline, recover physical health and optimize function in the presence of age-related loss or disease [5]. People with higher resilience are expected to recover faster and to recover more often to their pre-fracture level of functioning, i.e. mobility and Activities of Daily Living (ADL). However, there is still limited knowledge regarding the role of resilience in the recovery trajectories of older adults after a hip fracture. Existing measurements to gain insight into resilience, such as the Barthel Index, and other surveys or physical tests, are often static or impossible to perform while being bedridden, making them not suitable for older adults shortly after hip fracture surgery [6]. Therefore, clinicians have to estimate based on intuition rather than on objective measurements whether older adults can resist or recover from health stressors such as hip fracture surgery [7]. Insight into resilience can help identify high-risk patients for delayed or incomplete recovery of the pre-operative level of functioning, resulting in a better understanding of the expected recovery trajectory. This enables adjustment of the treatment plan when necessary, leading to personalized care.

Measurement tools have been developed to gain insight into resilience. One of these relatively new measures is fatigability, which can be divided into the self-perceived experience of fatigue and the resistance of physical tiredness, such as muscle fatigability [8]. Fatigability relates to the concept of physical resilience as it is understood to be a direct measure of ‘’the whole person’s response to stressors’’ [9, 10].

To determine fatigability, the Capacity to Perceived Vitality ratio (CPV), which represents the ratio of self-perceived fatigue (SPF) and muscle fatigability, can be used [8]. The SPF is used because fatigue represents a reduction in reserve capacity, which correlates with declines in intrinsic capacity, thereby increasing the likelihood of negative health outcomes [11]. SPF can be measured with a survey or single item questions. In addition, grip work (GW) is a dynamic measurement for muscle fatigability, which combines maximum hand grip strength (GS) with fatigue resistance (FR), here defined as the time a person can sustain maximum GS until 50% of the maximum strength has been reached [12–14]. Low GS is being used as one of the measures for frailty [15–18], and low FR has been correlated to frailty [18] and ADL dependency [13, 19]. As GW combines both GS and FR, it could be considered a measure of the dynamic physical reserve of the individual that enables resilient responses [13, 18]. Previous research showed that pre-frail older adults experience significantly lower CPV levels compared to non-frail older adults [9]. Pre-frailty is defined as a subclinical state of frailty. In addition, CPV ratios appear to have a stronger relationship with frailty than GW and SPF separately [9]. Because CPV, is presenting the ratio between GW and SPF it can be considered as a good instrument to identify subclinical fatigue and giving insight in resilience [9].

As previously mentioned, there is still little knowledge on resilience and its fundamental role in older adults during recovery after hip fracture surgery. Therefore, this study aims to assess whether fatigability, calculated with the CPV ratio, at baseline and its development over time can provide insight into the resilience of hip fracture patients. Additionally, this study aims to investigate whether CPV is related to short-term hospital discharge outcomes, such as mobility and the development of complications, and may thereby provide insight into recovery and resilience.

## Methodology

### Study design

In this prospective cohort study, patients were included from March 2022 until February 2024, with the aim of including 110 patients. The data used in this study were collected within the FORTO 2.0 project, with the data collection specifically designed to address the present sub-aim. This study was conducted according to the principles of the Declaration of Helsinki (75th WMA General Assembly, Helsinki, Finland, October 2024) and reviewed by the Medical Research Ethics Committees United (MEC-U) and the institutional review board of ZGT. The MEC-U stated that this study was subject to the Medical Research Involving Human Subjects Act (file number: NL78347.000.21). All patients gave written informed consent prior to participating in this study.

### Study population

All patients aged 70 years or older, who were surgically treated for a hip fracture, and admitted to the Center for Geriatric Traumatology (CvGT) at Ziekenhuisgroep Twente (ZGT) were invited to participate. Exclusion criteria for this study were: informed consent could not be obtained, being judged unable to participate by their treating physician, severe cognitive impairment, being physically unable to perform the GW measurements, being unable to communicate sufficiently in Dutch, deafness, a pathological or periprosthetic fracture, a total hip replacement and contact isolation. The treating physician was only consulted when there was uncertainty regarding the eligibility of a patient. Patients were included in the study on day one or two after hip fracture surgery.

## Study procedure

### Baseline variables

Baseline variables collected in this study during routine care were age, gender, pre-fracture living situation, pre-fracture Katz Index of Independence in Activities of Daily Living (KATZ-ADL 6), Pre-Fracture Mobility Score (PFMS), and Charlson Comorbidity Index (CCI).

Pre-fracture living situation was categorized as independent without home care services, independent with home care services, nursing home, geriatric rehabilitation centre, care home, and other living situations. The KATZ-ADL 6 assesses the independence of patients in ADL, with a score ranging from 0 (ADL independent) to 6 (completely ADL dependent) [20]. The PFMS classifies the patient’s mobility ranging from 1 (freely mobile without aids) to 5 (no functional mobility using lower limbs) [21]. The CCI predicts the 10-year survival in patients with multiple comorbidities, where a higher score corresponds to a higher burden of comorbidity [22].

### Maximal grip strength (GS), fatigue resistance (FR) and grip work (GW)

Each assessment of GW followed the protocol as introduced by Bautmans et al. using the Eforto® vigorimeter [12]. The Eforto® vigorimeter is an instrument designed to assess muscle fatigability and allows assessment of GW in older adults. In addition to the Eforto® vigorimeter, a smartphone-based application was used, which connected to the Eforto® vigorimeter via Bluetooth. A web platform was used to view the collected data. Each GW measurement was performed with the dominant hand and started with three separate GS measurements, with a rest period of 30 s between successive attempts. FR (seconds) was subsequently measured by the FR test [12], where patients were asked to squeeze the Eforto® vigorimeter bulb as hard as possible and were motivated to maintain their maximum GS as long as possible. The time (seconds) it took for the GS to drop below 50% of the maximum GS was defined as the FR [23]. To determine the maximal GS (kPa), the highest of three maximum GS attempts and the first 5 s of the FR test were used [12, 24]. The application automatically calculated GW by using the following formula: GW (in kPa*s) = maximum GS × 0.75 × FR [12]. The GW measurements took place twice daily, before noon and late in the afternoon, at the ward until hospital discharge. Measurements only took place when the patient was feeling good enough to follow instructions and was able to respond adequately to the questions. No measurements were performed during the weekend.

### Self-perceived fatigue

Prior to every GW measurement, SPF was measured using two single-item questions based on the Multidimensional Fatigue Inventory (MFI-20) survey. The MFI-20 survey collects information about self-perceived fatigue over the past few days [25]. A selection of two single-item questions was used because the MFI-20 asks questions about the past few days, which is not relevant when the measurement is taken twice a day. The following two questions were chosen to address both the trait and state of fatigue. The first question was: “*Hoe moe voelt u zich op dit moment?*” (How tired do you feel at this moment?) on a scale from 1 to 7 with 1 = “*niet moe*” (not tired at all) and 7 = ”*zeer moe*” (very tired) [8]. For the second question, the patient was asked to respond to the following statement “*Ik voel me gauw moe*” (I feel tired easily) by giving a number between 1 and 7. 1 is “*nee dat klopt niet*” (no, that is not correct) and 7 means “*ja dat klopt*” (yes, that is correct). These questions provided information about the individual’s levels of fatigue [26].

### Capacity to Perceived Vitality ratio [CPV]

Fatigability was calculated using the CPV ratio. This CPV ratio was calculated by the following formula: CPV (in points) = GW/(SPF question 1 + SPF question 2) [8, 9]. This resulted in higher CPV scores when GW was high and SPF was low, meaning these patients experience less fatigue and are more resistant to physical tiredness. When GW was low and SPF was high, this resulted in lower CPV scores, meaning these patients experience more fatigue and are less resistant to physical tiredness.

### Hospital discharge variables

At hospital discharge, the following variables were collected: Functional Ambulation Categories (FAC), Fracture Mobility Score (FMS), KATZ-ADL 6, length of hospital stay in days, discharge destination after hospitalization, complications during hospitalization, and delayed recovery (yes/no) due to a medical complication.

The FAC evaluates the patient’s degree of walking independence, ranging from 0 (non-functional) to 5 (independent, unrestricted) [27]. Based on these scores, the FAC variable in the present study was dichotomized into dependent on verbal or physical guidance (FAC < 4) and independent of verbal or physical guidance (FAC ≥ 4) which is in agreement with studies by Mehrholz et al. and Veerbeek et al. [27, 28]. Discharge destination was categorized as independent without home care services, independent with home care services, nursing home, geriatric rehabilitation centre, care home, and other destinations. Complications during hospitalization were measured as either present or not present and were assessed by the treating physician. Delayed recovery due to a medical complication was defined as a stay in the hospital that exceeds the expected discharge date (4 days after surgery) caused by a medical complication.

### Statistical analysis

The statistical analyses were performed using IBM SPSS Statistics (version 29). For all analyses, a statistical threshold of α = 0.05 was used. Continuous variables were presented as means (standard deviation; SD) in a normal distribution and as medians (interquartile range; IQR) in a non-normal distribution. Categorical variables were presented as numbers and percentages. Patients with an insufficient number of CPV measurements (less than three) were excluded from the following analyses. If the CPV data were skewed, the log transformation of CPV was used in all analyses.

The distribution and range of all CPV measurements were first examined, along with the progression of CPV over time during the hospital stay for each patient. Subsequently, it was statistically tested whether there was a change in CPV over time during the hospital stay. A mixed-model analysis was conducted to assess CPV development over time. First, an unconditional means model (Model A) was conducted. This model provides a null model to evaluate the fit of subsequent (unconditional growth) models. Subsequently, three additional linear mixed models were conducted and compared to the null model (Model A) to see which of these three models fitted the data best. These three models differed based on their fixed and random effects, where fixed effects estimate the average effects across all patients, and random effects capture variability across patients, allowing for individual differences. The intercept is the expected value of CPV when all predictors are set to zero at baseline. The slope indicates the expected change of CPV associated with a one-unit increase in time (half-day).

The three models (also called unconditional growth models) included:

- Model B = with a fixed intercept, a random intercept, a fixed linear slope and a random linear slope
- Model C = with a fixed intercept, a random intercept, and a fixed linear slope: because this model has no random slope, all individually estimated curves were forced to follow the sample estimated slope
- Model D = with a fixed intercept, a random intercept, and a random slope: because this model had no fixed slope, the sample estimated slope was constrained to zero, but individual curves were allowed.

The − 2 log-likelihood (− 2LL) of Models B, C and D were used to determine which model fitted the data best compared to Model A, with a lower − 2LL indicating a better fit to the data. After the model with the best fit was selected, covariables were examined one by one for their association with intercept and slope. Covariables that were examined were age, gender, pre-fracture living situation, pre-fracture KATZ-ADL 6, PFMS, and CCI. Next, we added those covariables that had a *p* < 0.05 in the univariable analysis to the multivariable model. Because KATZ-ADL 6 had a right-skewed distribution, the KATZ-ADL 6 score was categorized as KATZ-ADL 6 score 0, KATZ-ADL 6 score 1 and KATZ-ADL 6 score 2 or more. In addition, age was centered on the sample mean.

Finally, two logistic regression models were used to assess the relation between the CPV development over time during the hospital stay and hip fracture recovery outcomes (i.e. mobility and development of complications). For this analysis, the estimated individual intercepts and slopes of CPV over time of the participants (extracted from the linear mixed model) were included as independent variables, and FAC score at discharge (dichotomized as described above) and complications (yes/no) during hospitalization were included as dependent variables. Age and gender were added to the logistic regression models.

## Results

### Participants

**Fig. 1:**
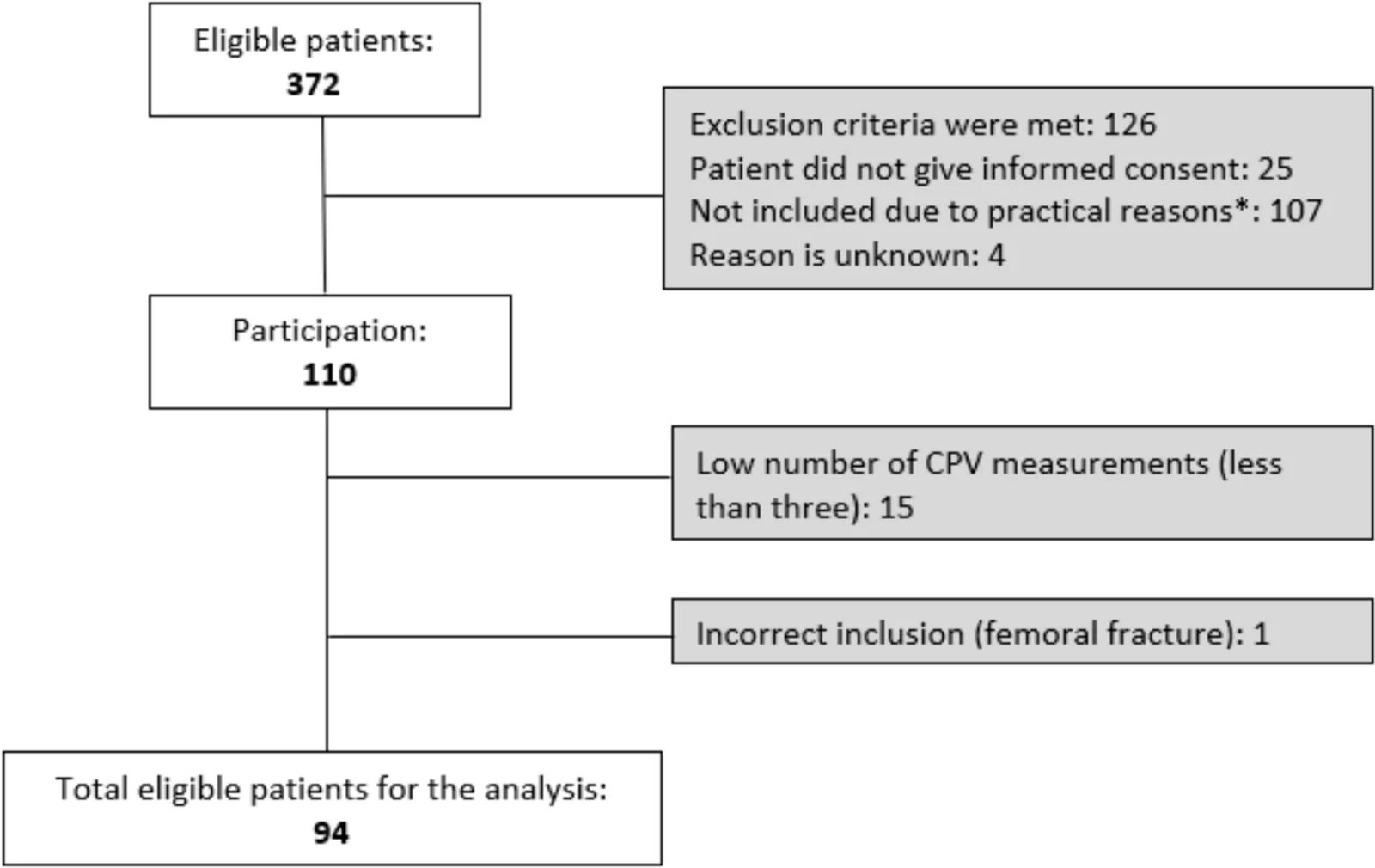
Flow chart. An overview of usable data. *Patients were not included during weekends or holidays

A total of 110 patients were included in this study. Additionally, fifteen patients were excluded due to a small number of CPV measurements. A small number of measurements was often due to the unavailability of the researcher. In addition, one patient was excluded because, after data collection, it was determined that the patient had a femoral fracture rather than a hip fracture. This resulted in 94 patients being included in the analysis of this study (Fig. 1).

The mean (SD) age of the patients was 81.8 (6.5) years old and 66% (*n* = 62) of the patients were female. A total of 69% (*n* = 64) of the patients scored a dependent FAC score at discharge (FAC score 0–3). In addition, the mean (SD) length of hospital stay was 7 (2) days and 30% *(n* = 28) of patients experienced a complication during hospitalization. The characteristics of the patients are presented in Table 1.

**Table 1:** Patient characteristics

Baseline variables	Total (*n* = 94)
Age (years); mean (SD)	81.8 (6.5)
Female gender; n (%)	62 (66.0)
Pre-fracture living situation; n (%)	
Independently without home care services	65 (69.1)
Independent with home care services	26 (27.7)
Nursing home (providing intensive care)	2 (2.1)
Geriatric Rehabilitation Centre	0 (0)
Care home (providing low-level care)	1 (1.1)
Other living situations	0 (0)
Pre-fracture KATZ-ADL 6 score; median (IQR)	0 (0–1)
PFMS; *n* (%)	
Freely mobile without aid	46 (48.9)
Mobile outdoors with one aid	5 (5.3)
Mobile outdoors with two aids or frame	40 (42.6)
Some indoor mobility but never goes outside without help	3 (3.2)
No functional mobility (using lower limbs)	0 (0)
Charlson Comorbidity Index (CCI); mean (SD)	6 (2)
**In-hospital variables**	
GW, first measurement at inclusion (kPa*s); median (IQR)	884 (523–1731)
Male	1186 (910–2167)
Female	668 (447–1388)
SPF, first measurement at inclusion; mean (SD)	
SPF question 1: “How tired do you feel at this moment?’’	4 (2)
SPF question 2: “I feel tired easily’’	4 (2)
CPV, at baseline (points); median (IQR)	120 (65–249)
**Hospital discharge variables**	
Complication during hospitalization; *n* (%)	28 (29.8)
Anemia	11 (11.7)
Delirium	6 (6.4)
Pneumonia	7 (7.4)
Heart failure	2 (2.1)
Kidney function disturbance	2 (2.1)
Other*	9 (9.7)
Day of first complication postoperative in days; mean (SD)	2 (2)
Length of hospital stay in days; mean (SD)	7 (2)
Delayed recovery due to a complication; n (%)	16 (17.0)
FAC at discharge, *n* (%)	
Independent (FAC score 4–5)	29 (31.2)
Dependent (FAC score 0–3)	64 (68.8)
KATZ-ADL 6 score, at discharge; median (IQR)	4 (2–4)
FMS, at discharge; *n* (%)	
Freely mobile without aid	0 (0)
Mobile outdoors with one aid	0 (0)
Mobile outdoors with two aids or frame	18 (19.4)
Some indoor mobility but never goes outside without help	67 (72.0)
No functional mobility (using lower limbs)	8 (8.6)
Discharge destination; *n* (%)	
Independent without home care services	5 (5.3)
Independent with home care services	17 (18.1)
Nursing home (providing intensive care)	1 (1.1)
Geriatric Rehabilitation Centre	68 (72.3)
Care home (providing low-level care)	1 (1.1)
Other destination	2 (2.1)

*SD,* standard deviation; *n,* number; *KATZ-ADL 6,* Katz Index of Independence in Activities of Daily Living; *IQR,* interquartile range; *(P)FMS,* (Pre-)Fracture Mobility Score; *CCI,* Charlson Comorbidity Index; *GW,* grip work; *kPa*, kilopascal; *s*, seconds; *SPF*, Self-Perceived Fatigue; *CPV*, Capacity to Perceived Vitality; *FAC*, Functional Ambulation Categories. * Consists of complications with an incidence of 1

### CPV measurements

Of a total of 999 CPV measurements that could have been executed if all of the twice-daily measurements were done as planned, 350 measurements were missing. The most common reason for a missing measurement was due to the fact that there were no measurements executed during the weekend (243, 70%). Other reasons for missing CPV measurements were: researcher unavailable (61, 17%), patient unavailable (25, 7%), patient declining to participate (18, 5%), patient could not be adequately instructed to perform the measurement (2, 0.6%), and patient deceased (1, 0.3%). In total, 649 CPV measurements were performed, with a mean (SD) of 7 (2) CPV measurements per patient. The CPV scores ranged from 6.5 points to 1990.2 points. Because CPV had a right-skewed distribution, the log transformation of CPV was used in the following analyses.

### CPV over time

To examine the CPV measurements over time, the first step was to determine which of the three unconditional growth models fit the data best by comparing the -2LL of Models B, C and D to Model A (-2LL Model A: 225.839; Model B: 195.700; Model C: 218.378; Model D: 198.328). Results of the difference in -2LL showed that the unconditional growth model (Model B) appeared to fit the data best and was used in the following analyses. In this model a fixed intercept, random intercept, fixed slope and random slope were allowed.

**Fig. 2:**
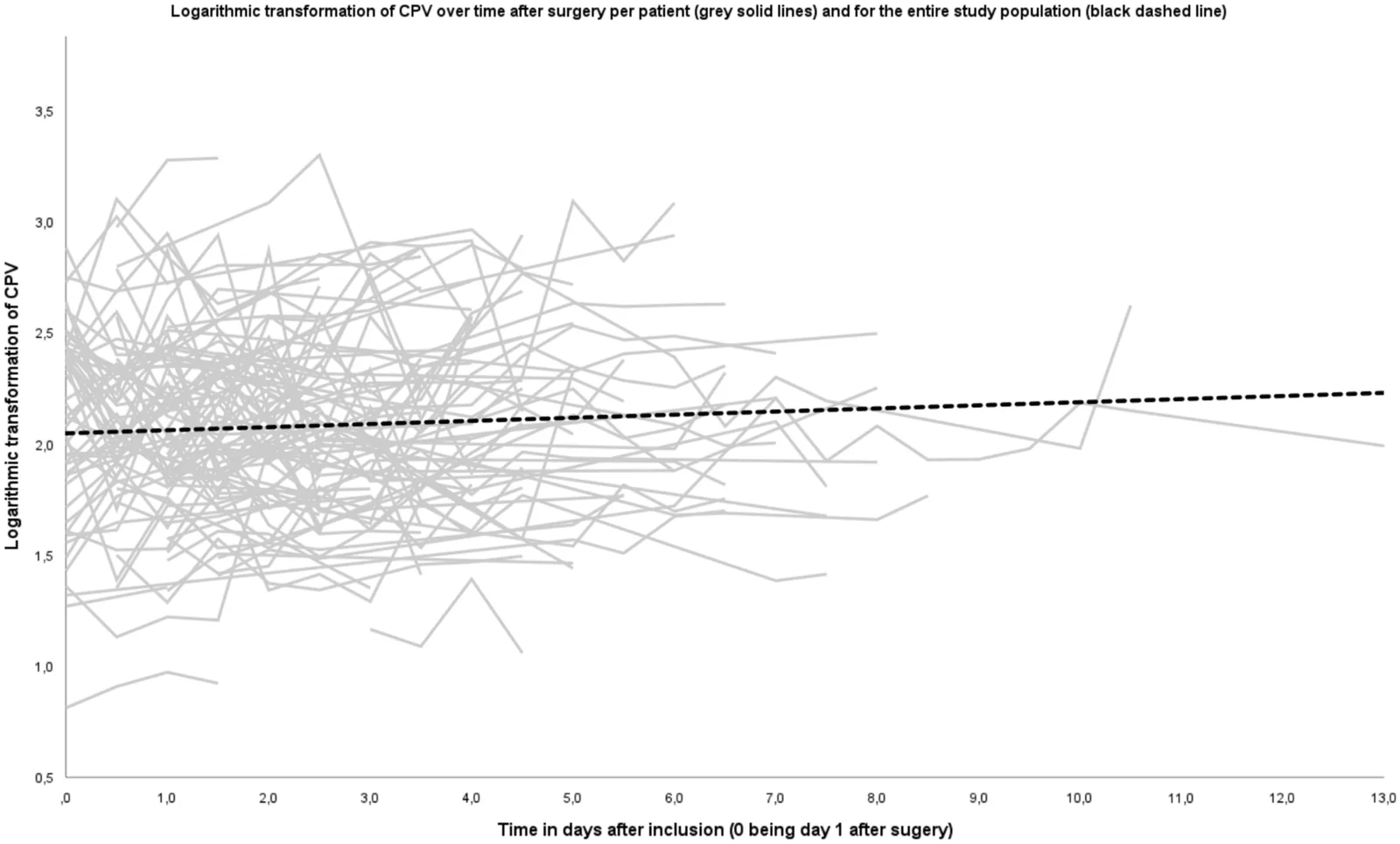
Logarithmic transformation of CPV over time after surgery per patient (grey solid lines) and for the entire study population (black dashed line). Equation of the black dashed line = 0,014 * x + 2,047

Figure 2 shows how LOG CPV developed over time (using Model B) for each patient (grey solid lines) and for the entire study population (black dashed line), where the day of inclusion is shown as day 0. CPV scores for the entire study population increased slightly over time (*p* = 0.11).

The covariables gender and categorized pre-fracture KATZ-ADL 6 showed a significant relationship with CPV and were therefore added to the model (Model B). The results of these two models can be found in Table 2.

**Table 2:** Unconditional and multivariable conditional growth model (model B) of CPV, back-transformed logarithmic numbers, over time with covariables gender and categorized pre-fracture KATZ-ADL 6

	Mixed model with random effects modelling					
	Unconditional growth model			Multivariable conditional growth model		
Parameter	Estimate	*p* value	95% Confidence Interval	Estimate	*p* value	95% Confidence Interval
Intercept	111.4	< 0.001	91.433–135.850	58	< 0.001	37.654–89.205
Gender						
Male				1.6	**0.01**	1.112–2.387
Female				Ref	–	–
Pre-fracture KATZ-ADL 6						
KATZ score 0				2.1	**0.003**	1.291 – 3.311
KATZ score 1				0.98	0.95	0.512 – 1.868
KATZ score 2 or more				Ref	–	–
Time	1	0.11	0.993–1.075	0.99	0.84	0.895 – 1.095
Gender						
Male * time				0.9	0.07	0.850–1.006
Female * time				Ref	–	–
Pre-fracture KATZ-ADL 6						
KATZ score 0 * time				1.1	0.11	0.980–1.217
KATZ score 1 * time				1.1	0.24	0.943–1.263
KATZ score 2 or more * time				Ref	–	–

For the covariables, gender and categorized pre-fracture KATZ-ADL 6, interactions with both the intercept and slope (time) were included in the model. Table 2 shows that patients with a pre-fracture KATZ-ADL 6 score of 0 had a factor 2.1 (*p* = 0.003) higher CPV scores at baseline than patients with a pre-fracture KATZ-ADL 6 score of 2 or more. At baseline, men had a factor 1.6 (*p* = 0.01) higher CPV scores than women. Over time, CPV did not change significantly (estimate = 0.99; *p* = 0.84). Table 2 also shows that the two covariables were not significantly associated with the slope (gender: *p* = 0.07; pre-fracture KATZ-ADL 6: *p* = 0.11 and *p* = 0.24). This means that the covariables influenced the intercept (between-person differences in CPV), but not the slope, and therefore not the CPV over time. However, the two covariables were close to significance with the slope. Meaning that men appeared to score a factor 0.9 lower CPV than women over time. Additionally, patients with a pre-fracture KATZ-ADL 6 score of 0 show a slight increase in CPV during the hospital stay compared to patients with a pre-fracture KATZ-ADL 6 of 2 or more.

### CPV at baseline

Table 3 presents both the univariable and multivariable logistic regression models, with FAC score at discharge and complications during hospitalization as dependent variables. The odds ratios of the intercepts and slopes in the logistic regression analyses were presented per one SD increase in CPV intercept and slope because a one-unit change on the original scale was considered too large or difficult to interpret, thereby simplifying the interpretation. Results of the first logistic regression model show that when the intercept of CPV increased by one SD, the odds of a dependent FAC score at discharge decreased significantly by a factor of 0.2 (*p* = 0.002) (Table 3). This indicates that patients with a higher CPV score at baseline had lower odds of a dependent FAC score at hospital discharge. In addition, the association between age and FAC score at discharge showed that the odds of a dependent FAC score at discharge increased by a factor of 1.2 (*p* < 0.001) when age increased by 1 year. Furthermore, it can be seen that when the slope of CPV increased by one SD, the odds of a dependent FAC score at discharge decreased by a factor of 0.7 (*p* = 0.13). However, this decrease was not statistically significant.

**Table 3:** Logistic regression analysis with dependent variables dichotomous FAC score at discharge and complication during hospitalization and independent variables intercepts and slopes of CPV over time of the participants, age, and gender. Odds ratios for intercept and slope are shown for an increase in CPV by one standard deviation

Parameter	Univariable		Multivariable	
	OR	*p* value	OR	*p* value
FAC score at discharge				
Age	1.2	** < 0.001**	1.2	** < 0.001**
Gender	1.3	0.53	1.1	0.88
Intercept	0.3	0.005	0.2	**0.002**
Slope	0.9	0.71	0.7	0.13
Constant			2860.8	0.04
Complication during hospitalization				
Age	1	0.54	1	0.74
Gender	0.6	0.24	0.6	0.23
Intercept	0.3	0.035	0.3	**0.037**
Slope	1.1	0.63	0.9	0.8
Constant			3.5	0.7

The results of the second logistic regression show that when the intercept of CPV increased by one SD, the odds of developing a complication during hospitalization decreased significantly by a factor of 0.3 (*p* = 0.037) (Table 3). This indicates that patients with a higher CPV score at baseline had lower odds of developing a complication during hospitalization. Furthermore, it can be seen that when the slope of CPV increased by one SD, the odds of developing a complication during hospitalization decreased by a factor of 0.9 (*p* = 0.80).

## Discussion

The aim of this study was to assess whether fatigability, calculated with the CPV ratio, at baseline and its development over time can provide insight into the resilience of hip fracture patients. Additionally, this study aimed to investigate whether CPV is related to short-term hospital discharge outcomes, such as mobility and the development of complications, and may thereby provide insight into recovery and resilience. The results showed that over time there was no significant change in CPV. In addition, the covariables gender and pre-fracture KATZ-ADL 6 were significantly related to CPV at baseline. Furthermore, the results show that a higher CPV ratio at baseline was associated with significantly lower odds of a dependent FAC score at discharge and of developing a complication during hospitalization. This shows that the CPV ratio at baseline may contribute to predicting the (in)dependence in mobility at discharge and whether or not a complication will occur during hospitalization.

To the best of our knowledge, no prior research has investigated CPV (or its two components, GW or SPF) by examining the development of CPV measurements over time during hospitalization in older adults following hip fracture surgery. We found that the covariables gender and pre-fracture KATZ-ADL 6 were significantly associated with between-person differences in CPV. This finding aligns with previous research by Knoop et al. showing that pre-frail men achieve better GW and CPV ratios compared to women [9]. In addition, previous research has shown that GW and GS were significantly related to independence in ADL in hospitalized geriatric patients [12, 29]. Furthermore, no research has been conducted on CPV at baseline and its association with short-term functional outcomes at hospital discharge. However, studies have been carried out on the components of CPV and functional outcomes. Research by Bautmans et al. has shown that GW is positively related to mobility in older nursing home residents [30]. In addition to these results, the present study demonstrates that CPV at baseline is associated with mobility at discharge, indicating that CPV could predict future mobility. This finding has clinical value and provides insight into recovery, thereby creating opportunities for personalized care. Also, results from a study on GS and functional recovery after hip fracture surgery indicate that GS was an indicator of functional status at admission and of functional recovery in the subsequent three months [31]. Finally, our results showed that a better CPV at baseline was associated with lower odds of developing complications during hospitalization. This partly aligns with previous research demonstrating that GS was associated with a decreased risk of complications during hospital admission and within 30 days thereafter in a population of adults with vascular diseases [32]. In addition, Bohannon’s study showed that hip fracture patients with weak grip strength were more likely to experience complications during hospitalization [18].

There was a large variation in CPV scores, with a minimum score of 6.5 points and a maximum score of 1990.2 points. However, it is not yet known what the meaning of this distribution is. The combination of GW and SPF to determine CPV used in the current study is a new method that, to the best of our knowledge, has not been investigated before. The CPV ratio has been used before, using the MFI-20 survey as a measure for SPF. However, these previous studies used a single measurement of CPV rather than multiple measurements over time [9, 33]. Because the MFI-20 asks questions about the past few days, it was unsuitable in the current study where SPF measurements were taken twice daily. Therefore, two SPF questions were used in the CPV ratio instead. This is partly in line with the study by Hoekstra et al., in which the single item question: “How tired do you feel at this moment?” was used in the CPV ratio [8]. Interchangeability of the results of studies using the CPV ratio can be increased by standardizing the CPV ratio. Research has been done into the reference values of grip strength in healthy adults between 20 and 95 years of age [34]. Further research is needed to identify reference values of grip work or CPV in older adults.

There was no significant average change in CPV scores over time. This suggests that measuring CPV twice a day does not provide additional value for determining trends during the relatively short period of the hospital stay. For this reason, it could be suggested that a single CPV measurement may be sufficient for this patient population. The mixed model shows that the added covariables gender and pre-fracture KATZ-ADL 6 were significantly associated with the intercept, but not with the slope. This means that the covariables have little influence on the within-person changes over time in the CPV measurements, and are mainly important for the between-person differences of CPV at baseline. An explanation for the little influence of the covariables on the CPV measurements over time could be that the follow-up interval was relatively short (a hospital stay with an average of seven days) in this study and there was no average change in the CPV measurements during that short period. Therefore, in the context of the present study and depending on the purpose of the measurement, we recommend the use of a single CPV measurement that does not necessarily need to be performed on a fixed postoperative day, for future research.

Approximately one-third of the CPV measurements were missing. The majority of these measurements were missing because of the non-availability of a researcher, especially during the weekend. This can be solved in the future by having the measurements performed as part of clinical care, and by conducting them less frequently [35, 36]. In addition, there were still 46 missing measurements. Since this is a small percentage (4.6%) of the total measurements that could have been executed (999), the Eforto® vigorimeter measurements seem to be an accessible way to measure GW over a longer period of time within a hospital in the context of the study. For example, to use it for monitoring patients to gain insight into mobility at discharge and complications during hospitalization. This corresponds to the article by Swart et al. which states that the GW measurements with the Eforto® vigorimeter were feasible, doable and easy to perform for most participants [36].

### Strengths and limitations

A strength of this study is that, to the best of our knowledge, no previous research has examined CPV over time for providing insight into resilience. In addition, this study has a prospective design in a fairly large systematically recruited patient cohort, and there was little loss to follow-up of patients during hospitalization.

This study has several limitations. The first limitation was that patients with severe cognitive disorders did not participate in this study because they were unable to give informed consent. As a result, this patient population is not represented in the current study, making the results less generalizable to all older adults with a hip fracture. Patients with a cognitive disorder are an important patient population in which more patients experience complications and associated postoperative readmissions and mortality than patients with normal cognition [37]. In addition, a cognitive disorder could influence the recovery process and degree of recovery, as patients with cognitive disorders may have difficulty with being instructed, following advice and performing physical therapy exercises. Therefore, to improve generalizability of the findings to all older adults with a hip fracture, it is important that future research also includes patients with cognitive impairment. A second limitation was that the cohort of this study was recruited from one hospital, which may limit external validation. Furthermore, to minimize the risk of overfitting we adjusted only for age and gender when we explored the predictive value of CPV intercept and slope for the different outcomes of interest (FAC at discharge and complications). Further study in preferably larger samples is needed to validly explore the added predictive value of CPV-derived indicators over and beyond other clinical variables such as frailty markers.

Future research could be conducted with a larger patient population from multiple hospitals to increase external validation. In addition, research should be done into the threshold values and clinical relevance of CPV scores, which can contribute to the interpretation of the CPV scores and thereby to insight into the resilience of older adults after hip fracture surgery. Furthermore, future research should focus on other potential applications of CPV, such as measuring CPV in the emergency department. This would potentially allow for gaining insight into resilience prior to surgery, enabling CPV to contribute to the decision-making process regarding whether to perform surgery. Finally, future research should focus on simplifying the use of the CPV ratio to improve its applicability in daily clinical practice and on its additional predictive value over and beyond other variables that are collected. Based on the results of this study, this may involve exploring the use of one measurement instead of multiple measurements over time, as well as investigating the individual contributions of both components (grip work and self-perceived fatigue) of the CPV ratio.

## Conclusion

Based on this prospective cohort study, we can conclude that the CPV measurements at baseline had a predictive value for mobility at discharge and complications during hospitalization. Furthermore, there was no significant change in CPV scores over time, and the covariables gender and pre-fracture KATZ-ADL 6 mainly influenced the baseline CPV measurement. In addition, the Eforto® vigorimeter seems to be an accessible way to measure GW over a longer period of time. However, it could be suggested that a single CPV measurement may be sufficient for this patient population. These results contribute to the possibility of gaining insight into resilience in an objective and systematic way and to the ability to estimate the degree of recovery at hospital discharge by using the CPV ratio.

## Data Availability

The data that support the findings of this study are available from the corresponding author upon reasonable request.
